# Acute Otitis Media-Induced Gradenigo Syndrome, a Dramatic Response to Intravenous Antibiotic 

**Published:** 2017-05

**Authors:** Tayebeh Kazemi

**Affiliations:** 1 *Otolaryngology Research Center, School of Medicine, Shiraz University of Medical Sciences, Shiraz, Iran.*

**Keywords:** Acute otitis media, Gradenigo syndrome, Medical therapeutics, Petrositis, Surgical intervention

## Abstract

**Introduction:**

Petrositis is a rare but severe complication of acute otitis media and mastoiditis. Despite efficient antibiotic therapy, there are still reports of both intratemporal and intracranial complications of otitis media with the potential risk of high morbidity and mortality. Petrositis has traditionally been treated with surgery, but recent advances in imaging, with improved antibiotic treatment, allow more conservative management.

**Case Report:**

In this case report we describe the clinical course and treatment of a 33-year-old man with petrous apicitis who presented with severe otalgia, retro-orbital pain, and sixth cranial nerve palsy Gradenigo syndrome. Our patient showed a dramatic response to intravenous antibiotics only, without need for any surgical intervention, even myringotomy.

**Conclusion:**

It seems that early detection and management of this syndrome before development of other intratemporal or intracranial complications may prevent the need for surgical intervention.

## Introduction

Petrositis is a rare and severe complication of acute otitis media (AOM) and mastoiditis (1,2). In Goldstein’ study of 100 children admitted to the Children's Hospital of Pittsburgh between 1980 and 1995 with an intratemporal complications of AOM, only four patients presented with acute petrositis (3). Each petrous apex is either under developed (sclerotic), contains marrow, or exhibits a variable degree of pneumatization. Pneumatization develops in only approximately 30% of temporal bones. 

Petrous apicitis is essentially mastoiditis that occurs in the petrous apex. Petrous apicitis can produce a triad of retro-orbital pain, sixth cranial nerve paralysis, and otorrhea, which has become known as Gradenigo’s syndrome. Only a minority of patients with petrous apicitis exhibit the full triad today (4). A review of the English-language literature for Gradenigo syndrome or petrous apicitis revealed only 48 publications concerning this condition, most in the otolaryngology press (5).

 Petrous apicitis is an extension of infection from the mastoid air cell tract into a pneumatized anterior or posterior petrous apex (6). Most of the Gradenigo syndrome cases that have been reported in the literature were caused by pyogenic bacteria, although there is a report documenting infections of nontuberculous mycobacteria (NTM) in Gradenigo syndrome. Thus NTM infection must be considered in chronic otomastoiditis complicated by Gradenigo syndrome not responding to initial broad-spectrum antibiotic therapy (7). 

A case of Aspergillus petrous apicitis was also reported (8). In the pre-antibiotic era, the incidence of intracranial complications of ear disease has been quoted at 2.3–6.4%, with a reduction to 0.04–0.15% due to the introduction of the widespread use of antibiotics (9). Sometimes extracranial complications of otitis media may be accompanied by intracranial complications such as meningitis and dural sinus thrombosis and cavernous sinus thrombosis (10,11).

Because of the anatomic complexities and the necessity to work around the labyrinth and the carotid artery, petrous apex air cell disease cannot be excised. Established drainage and prolonged antibiotics are an integral part of treatment, with or without surgery (4). Petrous apicitis has traditionally been treated with aggressive surgical methods. However, recent reports describe good results with more conservative medical treatment and minimal surgical intervention (12).

We present a case of petrositis after AOM with abducens nerve palsy and retro-orbital pain, which was managed medically with only IV antibiotics and steroids without the need for surgical intervention, even myringotomy.

## Case Report

Our patient was a 33-year-old male, who initially had presented to the neurologic clinic with left-sided lateral gaze restriction, horizontal diplopia, and retro-orbital pain for 8 days, but with no history of photophobia. The patient also had an 8-week history of left-sided otalgia that improved partially after receiving a 5-day course of oral azithromycin, but became aggravated again 3 days prior to admission. The patient’s hearing status was not changed subjectively. In terms of past medical history, the patient had no complaint of otorrhea or ear problems. 

He had a history of head injury with an altered level of consciousness the previous year, improving after 3 days. On admission, a physical examination showed normal vital signs (including normal body temperature) with a left-sided red bulge on the tympanic membrane (TM), but no evidence of perforation, otorrhea, retro-auricular swelling or tenderness was noted. Eye movement was restricted in the left-sided lateral gaze, but corneal sensation was normal. The patient had normal symmetrical facial movement bilaterally. Other cranial nerves were also intact.

**Fig 1:**
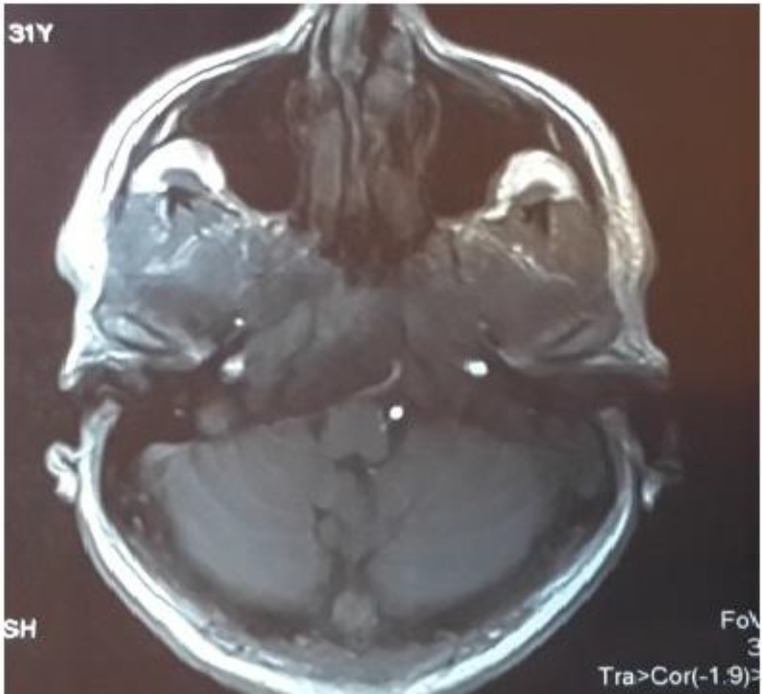
Brain MRI axial T1WI without Gad showing left mastoid air cell opacification and involvement of the left petrous apex

**Fig 2:**
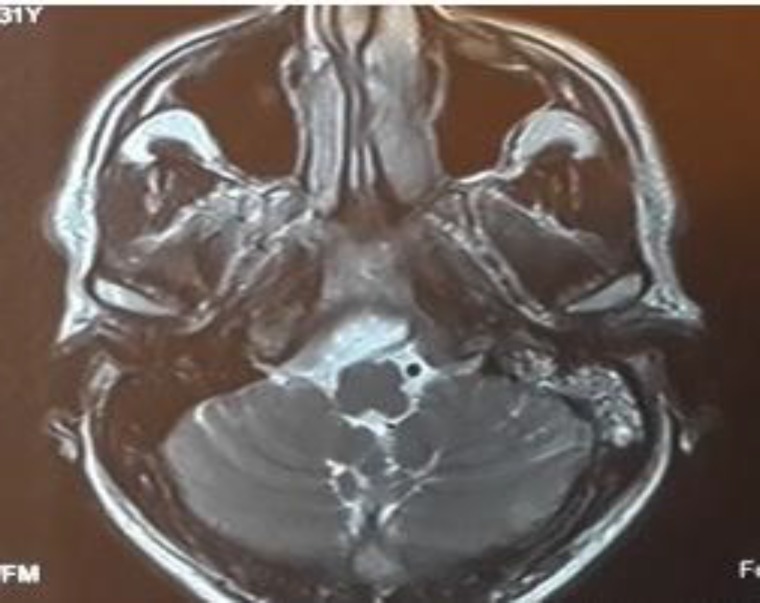
Brain MRI axial T2WI without Gad showing left mastoid air cell opacification and involvement of the left petrous apex

**Fig 3:**
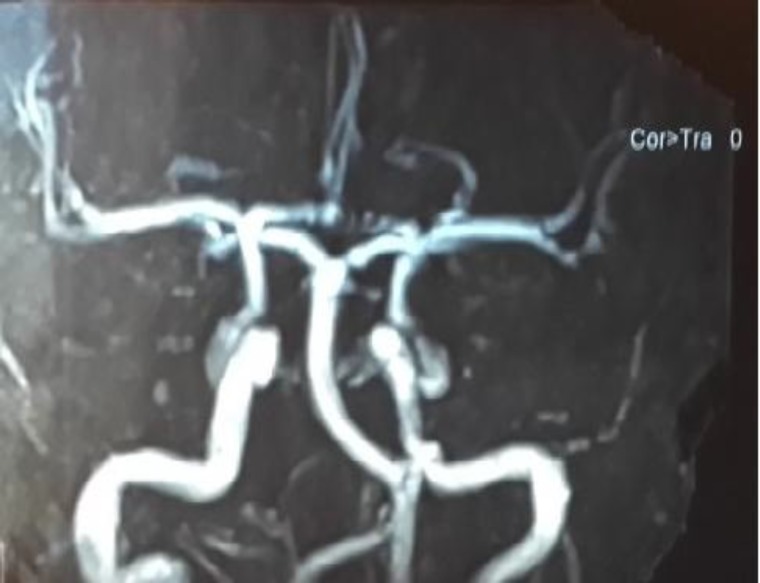
MR venography with contrast: No evidence of lateral sinus thrombosis is seen

Due to abducent nerve palsy and gaze restriction, the patient was initially evaluated by a neurologist, and a brain magnetic resonance imaging (MRI) scan was taken. Because of MRI evidence of mastoiditis and petrositis, the patient was referred to our clinic for further evaluation and management (Fig.1,2). In concurrent MR venography, no evidence of lateral sinus thrombosis was detected (Fig.3). At that time our computed tomography (CT) scanner was not working, so a high-resolution CT scan was not performed on this patient.

Laboratory tests showed a white blood count of 10.7 × 109/L; 57.8% neutrophil; an erythrocyte sedimentation rate of 10 mm/h and a negative C-reactive protein result. 

**Fig 4:**
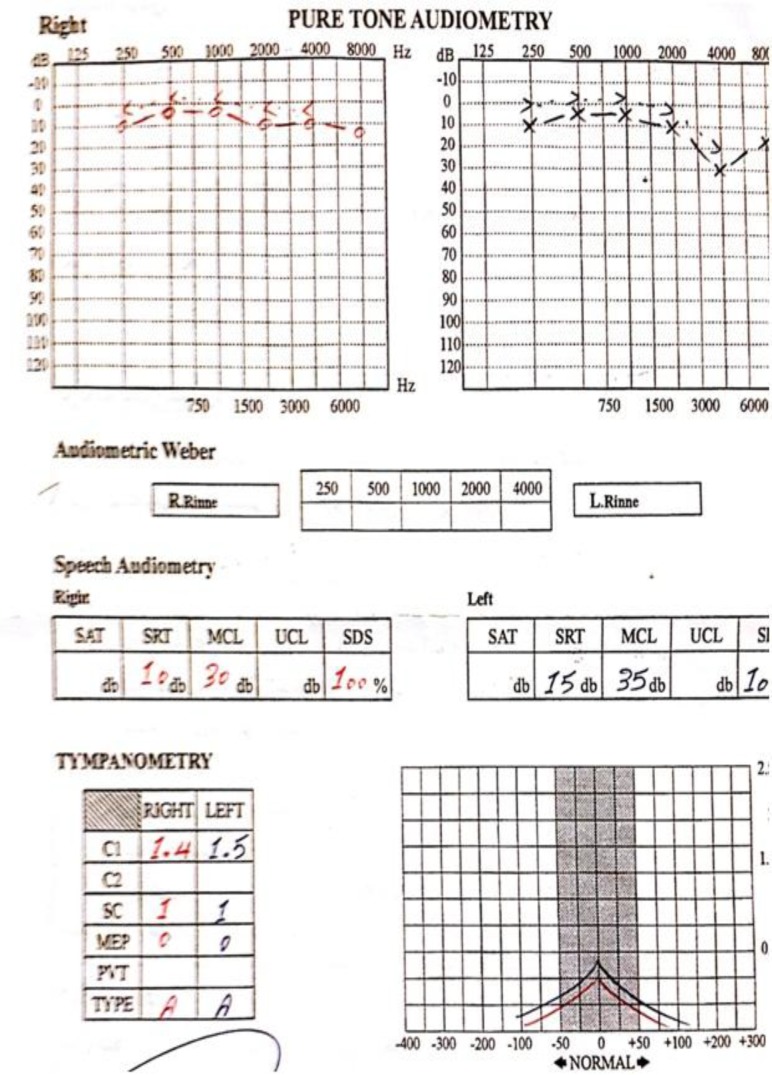
Audiometry, 2 months after admission, mild sensorineural hearing loss in 4 KH in left side

Pure tone audiometry performed 2 months later showed mild left-sided sensorineural hearing loss that peaked at 4 kHz, but the patient did not complain of recent subjective changes in hearing status. This mild hearing loss may have been due to a previous head injury (Fig.4).

The patient was treated with clindamycin 600 mg intravenously (IV) every 6 hours and ceftazidime 1 g IV every 8 hours for 5 days. In addition, the patient received dexamethasone 8 mg IV every 8 hours for 3 days. On the second day in our institution, otalgia was improved. One day later the patient had no complaint of retro-orbital pain, and on the fourth day after admission, the left lateral gaze returned to normal without any restriction. 

The patient was discharged on oral co-amoxiclav for 2 weeks. After 2 weeks of visiting the out-patient department, the patient was followed-up and found to have not complaints.

**Fig 5:**
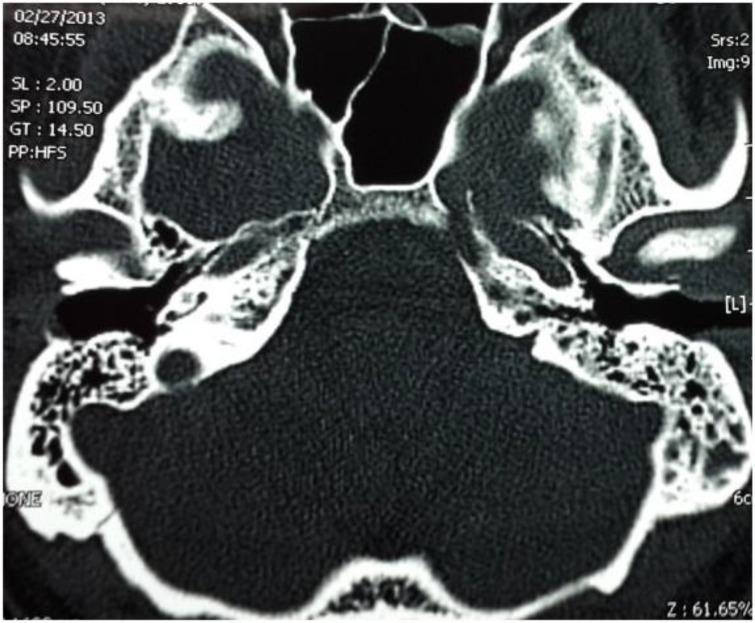
Axial temporal bone CT image showing disappearance of opacification and improvement of aeration at left petrous apex and mastoid bone

A follow-up temporal bone CT was taken approximately 6 weeks after discharge. This scan showed the disappearance of the left mastoid bone and petrous apex opacification with good aeration of both areas (Fig. 5).

## Discussion

Gradenigo syndrome is an uncommon but life-threatening complication of otitis media. The most common symptoms are deep facial or retro-orbital pain (from irritation of contiguous trigeminal ganglion in Meckel’s cave), or paralysis of the abducens nerve as it passes through Dorell’s canal abutting the petrous apex or dysfunction of facial or cochleovestibular nerves as they course through the temporal bone. The typical presentation of Gradenigo syndrome comprises retro-orbital pain, sixth cranial nerve palsy, and otorrhea. Only a minority of patients exhibit the full triad today (4). Our patient developed retro-orbital pain and abducens nerve palsy without any otorrhea, although otalgia was the initial complaint in his clinical course and at presentation he had overt AOM.

Petrous apicitis might develop following AOM or may occur in a patient with chronic otitis media. Typical causative organisms in AOM are Streptococcus pneumoniae, Haemophilus influenzae*, *and Branhamella catarrhalis, and a combination of aerobic and anaerobic organisms have been cultured from patients (4). Due to his previous history, our patient appeared to be a case of AOM that developed into Gradenigo syndrome. Because of his dramatic response to IV antibiotics, the patient did not need tympanocentesis or myringotomy and culture; therefore no causative organisms were identified.

The time interval between the onset of otitis media and the clinical presentation of abducens nerve palsy varies from 1 week to 2–3 months (13). Among our patients, this time is typically about 7 weeks.

Most reported cases of Gradenigo syndrome in the last decade have developed secondary to AOM in children (5,9,13,14). In the present case of this adult 33-year-old man, symptoms also followed an acute attack of otitis media.

Established drainage and prolonged antibiotics are an integral part of treatment, with or without surgery (4). Most authors advocate surgery, due to the possibility of fatal complications. Chole and Donald stated that aggressive surgical drainage is indicated when petrous apicitis is diagnosed (15) and Watkyn-Thomas reported that petrositis is curable by adequate mastoid operation (16). Hendersot et al. presented a middle fossa approach for the treatment of petrous apicitis (17). However, recent reports advocate conservative therapy with high-dose broad-spectrum antibiotics and less aggressive surgical procedures (5). Indeed, five cases among 48 reviewed from the English-language literature were treated without surgery (6). In our case, a dramatic response was achieved by administering IV antibiotics for 3–4 days without the need for surgical intervention, even a ventilation tube. Our patient was discharged after a 5-day course of IV antibiotic therapy.

In a case reported by Scardapane, complete resolution of symptoms and radiological alterations were observed within 7 weeks (13). In the present case, all clinical signs and symptoms were resolved during the first 4 days of admission. Complete resolution of mastoid and petrous bone opacification was seen on a high-resolution CT scan of the temporal bone performed 6 weeks after discharge.

## Conclusion

In conclusion, we emphasize the possibility of the conservative management of petrositis. This is not the first report of Gradenigo syndrome complicating a case of otitis media. However, our case is a good example of successful and dramatic medical resolution due to early diagnosis and adequate prompt treatment with IV antibiotics before development of other intratemporal or intracranial complications. Clearly, when conservative therapy fails or chronic ear disease is present, surgical intervention is indicated to ensure adequate drainage of all mastoid and petrous bone air cells.
